# Molecular Mechanisms for cAMP-Mediated Immunoregulation in T cells – Role of Anchored Protein Kinase A Signaling Units

**DOI:** 10.3389/fimmu.2016.00222

**Published:** 2016-06-08

**Authors:** Vanessa L. Wehbi, Kjetil Taskén

**Affiliations:** ^1^Nordic EMBL Partnership, Centre for Molecular Medicine Norway, Oslo University Hospital, University of Oslo, Oslo, Norway; ^2^Jebsen Inflammation Research Centre, Oslo University Hospital, Oslo, Norway; ^3^Biotechnology Centre, Oslo University Hospital, University of Oslo, Oslo, Norway; ^4^Jebsen Centre for Cancer Immunotherapy, Oslo University Hospital, Oslo, Norway; ^5^Department of Infectious Diseases, Oslo University Hospital, Oslo, Norway

**Keywords:** cAMP, AKAP, protein–protein interaction, T cell, prostaglandin

## Abstract

The cyclic AMP/protein kinase A (cAMP/PKA) pathway is one of the most common and versatile signal pathways in eukaryotic cells. A-kinase anchoring proteins (AKAPs) target PKA to specific substrates and distinct subcellular compartments providing spatial and temporal specificity for mediation of biological effects channeled through the cAMP/PKA pathway. In the immune system, cAMP is a potent negative regulator of T cell receptor-mediated activation of effector T cells (Teff) acting through a proximal PKA/Csk/Lck pathway anchored via a scaffold consisting of the AKAP Ezrin holding PKA, the linker protein EBP50, and the anchoring protein phosphoprotein associated with glycosphingolipid-enriched microdomains holding Csk. As PKA activates Csk and Csk inhibits Lck, this pathway in response to cAMP shuts down proximal T cell activation. This immunomodulating pathway in Teff mediates clinically important responses to regulatory T cell (Treg) suppression and inflammatory mediators, such as prostaglandins (PGs), adrenergic stimuli, adenosine, and a number of other ligands. A major inducer of T cell cAMP levels is PG E_2_ (PGE_2_) acting through EP2 and EP4 prostanoid receptors. PGE_2_ plays a crucial role in the normal physiological control of immune homeostasis as well as in inflammation and cancer immune evasion. Peripherally induced Tregs express cyclooxygenase-2, secrete PGE_2_, and elicit the immunosuppressive cAMP pathway in Teff as one tumor immune evasion mechanism. Moreover, a cAMP increase can also be induced by indirect mechanisms, such as intercellular transfer between T cells. Indeed, Treg, known to have elevated levels of intracellular cAMP, may mediate their suppressive function by transferring cAMP to Teff through gap junctions, which we speculate could also be regulated by PKA/AKAP complexes. In this review, we present an updated overview on the influence of cAMP-mediated immunoregulatory mechanisms acting through localized cAMP signaling and the therapeutical increasing prospects of AKAPs disruptors in T-cell immune function.

## Introduction

Cyclic AMP (cAMP) is a second messenger, which relays signals from the outside to the inside of a cell and triggers downstream signaling cascades. Modulation of the intracellular cAMP concentration reflects changes in the cellular environment and creates changes in cellular function. In the T cell, cAMP is known as a potent negative regulator, which dampens T-cell immune function through the cAMP/protein kinase A (PKA) signaling pathway. Indeed, the cAMP/type I PKA/Csk/Lck [lymphocyte-specific protein tyrosine kinase (PTK)] signaling module has been defined as a dominant regulator driving the inhibition of T-cell function (1).

Specificity of cAMP signaling is achieved by compartmentalization through A-kinase anchoring proteins (AKAPs). Most cells express between 10 and 15 different AKAPs (2) and so far 7 AKAPs have been identified in T cells. AKAPs are characterized by their cellular localization mediated through a targeting domain and their binding partners, which define the spatiotemporal control of cAMP signaling, the interaction with other signaling pathways, and contribute to distinct cellular functions. In T cells ezrin functions as an AKAP and assembles a supramolecular signaling complex with PKA type I, EBP50 [ezrin–radixin–moesin (ERM)-binding phosphoprotein 50], phosphoprotein associated with glycosphingolipid-enriched microdomains (PAG), and Csk in the vicinity of the T cell receptor (TCR), which in turn modulates T cell immune responses (3).

The cAMP/PKA inhibitory signaling pathway controlling TCR signaling plays a key role in maintaining homeostasis in the T cell. However, any imbalance in TCR regulation can lead to T cell dysfunction and dramatic functional consequences. During diseases, such as cancer and chronic infections, T cells have high cAMP concentrations, which in turn cause excessive downregulation of TCR signaling and can favor disease development. Regulation of the cAMP/PKA pathway is crucial to protect against inappropriate regulation and immunological overshoot. Non-steroidal anti-inflammatory drugs (NSAIDs) are known to negatively regulate this pathway through their inhibitory action on the activity of cyclooxygenases (COX). NSAIDs, aspirin, and coxibs (selective COX-2 inhibitors) block prostaglandin E_2_ (PGE_2_) synthesis, which in turn downregulates the intracellular cAMP concentration in T cells. A number of studies have agreed on the beneficial use of COX inhibitors to enhance anti-tumor responses cancer (4–7). Despite their efficiency, the broad-spectrum activity of COX inhibitors can trigger unwanted effects, which may be avoided with new drugs that target the pathway at a different level. Because AKAPs scaffold supramolecular complexes acting as signal processing hubs that coordinate multiple signals in normal and aberrant conditions, protein–protein interaction disruptors that displace particular components of such complexes emerge as essential research tools and potentially targeted drugs complementary to the current therapeutic strategies.

## Molecular Mechanisms of Immunoregulation in T Cells: cAMP/PKA/Csk Pathway

Cyclic AMP is an intracellular second messenger (8) identified by the Nobel-Prize winning work of Earl Sutherland (9), able to trigger a plethora of signaling pathways leading to different biological outcomes. In effector T cells (Teff), PGs (10), adenosine (11), histamine (12), β-adrenergic agonists (13), neuropeptide hormones (14), and β-endorphin (15) induce cAMP, which acts as a potent negative regulator of TCR-mediated activation and proliferation (16–18). This contributes to regulation and maintenance of a healthy immune response. Any imbalance in regulatory mechanisms creates immune disorders and can lead to autoimmune diseases, chronic inflammation, and allergic responses.

### Prostaglandin E_2_ as a Potent Immunosuppressor

Prostaglandin E_2_ is the most ubiquitous PG produced by the human body and plays a critical role in guiding and governing various aspects of the inflammatory response. The role of PGE_2_ in driving acute inflammation is well established. However, PGE_2_ also elicits powerful immunosuppressive properties that contribute to the resolution phase of acute inflammation, the tissue regeneration, and the return into homeostasis. These multifaceted properties of PGE_2_ are both cell-type- and context-specific. The production of PGs begins with the liberation of arachidonic acid from membrane phospholipids by phospholipase A_2_ in response to inflammatory stimuli. Arachidonic acid is converted to PGH_2_ by the COX enzymes COX-1 and COX-2, and then to PGE_2_ by cell-specific PG synthases. Whereas COX-1 is considered as a ubiquitous housekeeping enzyme constitutively expressed and responsible for maintaining basal PG levels important for tissue homeostasis, COX-2 is an inducible enzyme that produces PGs during inflammatory conditions (19, 20). In tumor cells, COX-2 is often overexpressed, which in turn upregulates PGE_2_ and contributes to the immune evasion and cancer immunotherapy resistance creating an environment rich in IL-10 and TGF-β, cytokines known to promote regulatory T cells (Tregs) differentiation and proliferation (21–29). Treg are known as a unique population of T cells that maintain peripheral immune tolerance and effectively inhibit autoreactive T cells (30–32) and Teff responses, such as cytokine production and proliferation (33). Treg produce and respond to PGE_2_, which acts as an autocrine factor and increases intracellular cAMP that in turn upregulates forkhead/winged helix transcription factor (FOXP3) expression. PGE_2_ enhances Treg induction and differentiation through FOXP3 upregulation (4, 34–38). Tregs have also been shown to have high endogenous cAMP levels, which can be explained by a FOXP3-dependent downregulation of phosphodiesterase 3 (PDE3 is known to decline cAMP levels) (39) and an adenylyl cyclase (AC) 9 upregulation (AC9 is known to synthetize cAMP) (40). Immunosuppressive activity can be mediated by intercellular transfer of cAMP from Treg to Teff via gap junctions (GJ) presumably formed by Cx43, which is the connexin in T cells (41–43). cAMP leakage into Teff have been found to exhibit suppressive activity by enhancing the expression and nuclear function of a potent transcriptional inhibitor, inducible cAMP early repressor (ICER) and modulating the levels of surface-expressed cytotoxic T lymphocyte antigen-4 (CTLA-4) (44). In this case, the suppressive mechanisms triggered by cAMP transfer are cell-contact dependent; a close proximity between donor and recipient cells is required for the transfer of the second messenger.

Prostaglandin E_2_ can also act in a paracrine manner through direct binding and activation of the E-prostanoid (EP) family of G protein-coupled receptors. PGE_2_ can activate four subtypes of EP receptor at the T-cell surface, called EP1–EP4. Upon activation, EP receptor couples to specific G protein and activates specific G protein-dependent signaling pathways. The active EP1 receptor coupled to Gq protein increases phosphatidylinositol hydrolysis and intracellular Ca^2+^ through activation of phospholipase-C. The active EP3 receptor coupled to Gi protein leads to the AC inhibition and intracellular cAMP decrease. Only active EP2 and EP4 coupled to Gs protein lead to cAMP production through AC activation. This in turn activates PKA that next can activate the transcription factor cAMP response element-binding protein (CREB). CREB is known for its role in cell proliferation, differentiation, and survival (45). CREB has been shown to induce transcription of immune-related genes that possess a CRE element, including interleukin-2 (IL-2), IL-6, IL-10, tumor necrosis factor alpha (TNF-α), and COX-2 (46, 47). CREB plays a role in T-cell function (48) and also drives the generation and maintenance of Treg in a TGF-β/FoxP3-dependent manner (45, 49–53). The paracrine effect of PGE_2_ is clearly EP receptor subtype dependent, but it seems also to depend on the level of EP receptor cell surface expression. Indeed, during mucosal inflammation IL-2 secretion has been found to be negatively correlated to the EP4 expression (54).

Most studies on T cells have focused on CD4+ cells and showed the roles of PGE2 in the modulation of proliferation, apoptosis, and cytokine production. Less is known about the effect on CD8+ T cells, but PGE_2_ can inhibit CD8+ T-cell proliferation (55) and decrease the production of interferon γ (IFN-γ) (56). The impact of PGE_2_ in immune cells can also be cell-type-specific. Indeed PGE_2_ suppresses the function of macrophages, neutrophils, T helper 1 cells (Th1) cells, and natural killers, whereas it promotes T helper 2 cell (Th2), Treg, and T helper 17 cell (Th17) responses (57, 58). PGE_2_ has no effect on or enhances the production of Th2 cytokines, such as IL-4, IL-5, and IL-10, but inhibits drastically the production of Th1 cytokines, such as IFN-γ, TNF-α, and IL-2 (59–63). The induction of the Th2 response by PGE_2_ was found to be mainly cAMP-dependent (64) and recently the transcription factor, cAMP response element modulator (CREM), was characterized as a negative regulator of Th2 responses and a key factor in allergic asthma (65). In addition, PGE_2_ has been shown to promote T-cell anergy (66), maintain memory T-cell survival (67), and inhibit γδ T-cell cytotoxicity through a cAMP/PKA-dependent mechanism (68).

Inhibitory mechanisms triggered by PGE_2_ in T cells started to be unraveled in the late 1980s [reviewed in Ref. (20, 69–71)]. PGE_2_ triggers anti-proliferative effects through interference with IL-2-mediated gene expression (72, 73) and inhibition of IL-2 receptor expression (74, 75). PGE_2_ inhibits IL-2 gene transcription by downregulating the activation of its promoter. Indeed cAMP inhibits nuclear transcription of the human interleukin-2 gene by targeting two transcription factors: the nuclear factor of activated T cells (NFAT) and the activator protein 1 (AP1) contained in IL-2 promoter in human T cells (76). Furthermore, recently Rodriguez and co-authors found that elevated intracellular cAMP through a PKA-dependent pathway can disrupt IL-2R complex formation, Jak3 catalytic activity, and the ability to phosphorylate Stat5, resulting in a severe reduction in IL-2R signaling and T lymphocyte proliferation (77).

Despite the well-established molecular mechanisms unraveling cAMP inhibitory effect on T-cell function, new studies continue to fulfill our understanding with additional and/or alternative mechanisms. A recent study has described a potential new role for exchange protein directly activated by cAMP (EPAC) in the T-cell suppressive process. Downstream effectors of cAMP can be PKA dependent (78), cAMP-regulated ion channels (79, 80), and EPAC dependent (81, 82). In T cells, effects mediated by cAMP seem to be most likely through PKA activation, since EPAC and cAMP-gated channels appear to be expressed at low level in T cells (82). However, the cAMP suppressive effects on Teff have been showed to require both PKA and EPAC-dependent pathways. The involvement of EPAC in Teff suppression has been proved by the ability to mimic the cAMP response with an EPAC-selective cAMP analog, combined with the insensitivity of the cAMP response to inhibitors of PKA. The authors suggested that EPAC may function as an alternative effector to promote cAMP-dependent but PKA-independent Teff suppression (83).

Cyclic AMP is mostly known as an immunosuppressant, however, the regulation of T-cell activation is not the result of a linear control driven by cAMP gradients but the sum of competitive mechanisms processing in parallel of the cAMP immunosuppressant effects. Besides the Gs/AC/cAMP pathway, active EP2 and EP4 receptors trigger multiple signaling pathways, which can counteract the immunosuppressant effect of cAMP. In addition, full T-cell activation generally requires accompanying signals as CD28 co-stimulation inducing several specific signaling pathways as well. Yao and co-authors have shown that cAMP-mediated suppression of TCR signaling can be overcome by simultaneous activation of PI3-kinase through activation of EP2/EP4 and/or CD28. PGE_2_ promotes Th1 differentiation via synergistic amplification of IL-12 signaling by cAMP and PI3-kinase (84). These findings underscore the complexity of the regulation of T-cell function, which is overall the result of positive and negative events on the TCR signaling pathway.

### The TCR Signaling Pathway and TCR Activation

#### TCR Signaling Networks

Activation of T cells is a key step in adaptive immunity and requires the coordination and organization of the components of the TCR complex with its surrounding signaling molecules leading to TCR signaling events. The molecular process begins when the TCR identifies a peptide presented by the major histocompatibility complex (MHC) expressed on the surface of antigen-presenting cells (APCs) (85) and that binds with high affinity to the TCR. The part of the receptor that recognizes the large variety of antigens is a highly polymorphic heterodimer of α and β chains, which is associated with polypeptides γ, δ, ϵ, and ζ. All CD3 chains contain immunoreceptor tyrosine-based activation motifs (ITAMs) (86, 87). Activation is accompanied by formation of the immunological synapse (88) where lipid rafts (membrane microdomains enriched in cholesterol and sphingolipids) and Src-family tyrosine kinase (SFK) Lck accumulate (89–91). Signal transduction requires that at least two ITAMs are phosphorylated by Lck (92–94). Phosphorylated ITAMs of CD3 chains serve as docking and activation sites for Syk family kinase members as the zeta-chain-associated protein kinase 70 (ZAP-70), which in turn is phosphorylated and activated by Lck (95). ZAP-70 activity is essential in conventional T cells (CD4+, CD8+ T cells) but apparently not in Tregs (96). Activated ZAP-70 phosphorylates another membrane raft component: the transmembrane adaptor protein “linker for activation of T cells” (LAT) (97, 98). LAT is phosphorylated on multiple tyrosine residues that next form docking sites for other adapter proteins, such as SLP-76, Grb-2, and Gads, and enzymes, such as PI3K and PLCγ1 (98–101), and is essential for downstream TCR signaling. Lack of LAT expression uncouples TCR-proximal tyrosine phosphorylation from these downstream signaling cascades (100, 102). Ultimately, TCR activation triggers induction of gene expression. The nuclear factors NFκB, NFAT, AP1, and CREB are activated (103), which in turn promotes transcription of important genes for immune activation (104). TCR activation also triggers secretion of cytokines, such as IL-2, IL-4, IL-6, and IL-12 (105), and controls cytokine receptor expression (106), leading to qualitatively different intracellular responses. Moreover, TCR activation and the cytokine milieu synergically influence cell fate determination. Indeed, weak signals through the TCR trigger CD4+ T cells to differentiate to Th2 cells (107), whereas strong signals lead to Th1 cells differentiation (105, 106). By guiding lineage commitment, the cytokine environment and TCR activation also tune the immune response. Th1 cells produce IFNγ, TNF-α, and IL-2, promote cell-mediated immune responses, and control intracellular pathogen infections. Th2 cells produce IL-4, IL-5, IL-9, IL-10, and IL-13, promote humoral immune responses, and mediate resistance to parasites, such as helminths (105, 108–110).

The aggregation of TCR microclusters upon activation forms the central supramolecular activation cluster (cSMAC) (111, 112), which is part of the immunological synapse formed between a T cell and the APC (113). Co-receptors, such as CD4, CD8, and CD28, and signaling molecules co-aggregate in the cSMACs; and, overall, this creates an environment that is conducive to precise T-cell activation. Appropriate spatiotemporal localization of proteins is a key factor determining signaling activity. To this end, lipid rafts play a critical role (114–116); they serve as signaling platforms that contain several key signaling components involved also in TCR signaling, such as SFKs, transmembrane adaptor proteins, phosphatidylinositol bis-phosphate (PIP2), and G-proteins, and aggregate to form the C-SMAC and IS. Spatiotemporal changes in this well-organized molecular machinery modulate TCR signaling pathways and, therefore, affect T-cell function.

#### Downregulation of TCR Signaling: Molecular Mechanisms Involved in the cAMP–PKA–Csk Inhibitory Pathway in T Cells Lipid Rafts

**Figure 1:**
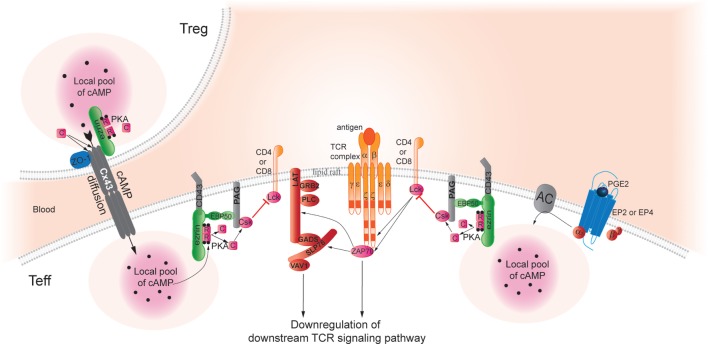
**Cyclic AMP immunoregulatory pathways inhibit TCR signaling and T-cell activation**. In Teff, pools of cAMP are generated after binding of PGE_2_ to its cognate receptors, which stimulates adenylyl cyclase (AC) activity and increases intracellular cAMP levels, thus, activating protein kinase A (PKA). Aided by an Ezrin/EBP50/PAG scaffold that holds both enzymes, PKA phosphorylates Csk, which in turn phosphorylates Lck to inhibit its activity. Lck normally acts to promote TCR signaling; thus, Lck inhibition through this PGE_2_-initiated pathway inhibits TCR signaling in effector T cells. Pools of cAMP can also be created by transfer from Treg to Teff through gap junctions (42). As a hypothetical model based on studies in trophoblasts (124), we speculate that this process may also require an AKAP bound to connexin 43 (Cx43) to facilitate PKA-mediated gap junction opening and cAMP transfer from Treg to Teff. Indeed, Pidoux and co-authors have found that ezrin binds to the C-terminal part of Cx43 and delivers PKA in the vicinity of gap junctions. Furthermore, the phosphorylation of Cx43 by PKA promotes opening of the gap junction and allows the passage of signal molecules. The authors suggested the PKA/ezrin/Cx43 macromolecular complex controlling the gap junction communication could be a general mechanism that regulates opening of Cx43 gap junctions in response to a cAMP increase also in other cell types. Thus, gap junctions may also deliver a local pool of cAMP that can dampen TCR signaling pathways by the same mechanisms described above and contributing to the Treg suppressive capacity of Teff.

Upon TCR activation, cAMP is rapidly produced in lipid rafts (117, 118) leading to the downregulation of TCR signaling, and then to the inhibition of T-cell proliferation and cytokine production. Several studies have unraveled molecular mechanisms involved in the inhibition of TCR signaling by cAMP and characterized the PTK, Csk, as a key player in TCR signaling downregulation (Figure 1) (1, 119–123).

Cyclic AMP serves as a second messenger within the cell and activates PKA, the dominant effector in T cells (82). The PKA is a heterotetrameric holoenzyme consisting of two regulatory (R) subunits that maintain two catalytic (C) subunits in an inactive state (125, 126). PKA exists in two classes, PKA type I and II that differ in the R subunit. Both R subunit and C subunit exist as multiple isoforms (RIα, RIβ, RIIα, RIIβ, Cα, Cβ, Cγ, and PrKX). The type I PKA is thought to be predominantly cytoplasmic and is most highly expressed in the immune system, whereas type II PKA associates with specific cellular structures and organelles and is abundant in the heart and the brain (127). When four molecules of cAMP bind its regulatory subunits, the PKA molecule releases the two catalytic subunits that have enzyme activity toward target proteins. Upon T-cell activation and formation of the immunological synapse, type I PKA is redistributed colocalizes with the TCR–CD3 complex (128). PKA type I phosphorylates the PTK, Csk, on serine 364 (1), which in turn initiates downregulation of the TCR signal. Indeed, Csk negatively regulates Lck by phosphorylation of a C-terminal inhibitory tyrosine residue, Y505 (129, 130) that contributes to stabilize Lck in an inactive conformation (131). Csk is recruited to membrane lipid rafts and is then activated (132) through its interaction with a transmembrane adaptor protein found in lipid rafts, PAG (133, 134), also known as Csk binding protein (Cbp). Upon TCR activation, PAG is dephosphorylated by a mechanism that appears to involve the activity of the phosphatase CD45 (135). Dissociation of Csk relieves Src kinases inhibition, enabling TCR downstream signaling pathways. Although Csk plays a critical role in the regulation of Lck activity, constant and abundant Lck fractions either phosphorylated on Y394, the active site, or double-phosphorylated on Y505 + Y394 reveals a more complex regulatory mechanism (136). Indeed, joint actions on phosphorylation state and spatial distribution of Lck are necessary for a balanced T-cell activity (137, 138).

#### Phosphodiesterases, Key Players in Modulation of T-Cell Signaling

The balance between the activities of two families of enzymes: ACs and cyclic nucleotide PDE regulate the intracellular cAMP level and its spatiotemporal distribution. PDEs are intimately coupled to limitation of cAMP gradients and termination of specific signals through local pools of cAMP and, therefore, multiple PDEs play important roles in modulating each cellular function [reviewed in Ref. (139, 140)].

Phosphodiesterases comprise a superfamily of enzymes classified into at least 11 families (PDE1–PDE11) and more than 50 isoforms that are distributed in different tissues at varying levels. PDEs have a highly conserved catalytic domain located near the C-terminus (>50% amino acid identity) flanked by regulatory domains in the N-terminus and the C-terminus. These family-specific regulatory domains include phosphorylation sites, membrane targeting domains, binding sites for small ligands, and dimerization domains [reviewed in Ref. (141, 142)]. Seven of the 11 families of PDEs have been reported to be present in T cells (143, 144). In most mammalian cells, PDE3 and PDE4 predominantly hydrolyze cAMP, and PDE4 is found as the major enzyme responsible for cAMP hydrolysis and dominant in inflammatory cells (145–147).

Phosphodiesterase 4 activity was detected in lipid rafts upon T-cell activation and especially after TCR and CD28 co-stimulation. Indeed, CD28 activation potentiates TCR signaling pathways and induces full TCR activation and clonal expansion (117, 148). Increase in PDE4 activity in response to CD28 co-ligation leads to negative regulation of the spatial and temporal cAMP gradient and thereby upregulates TCR signaling pathways. More specifically in human T cells PDE4A4, PDE4B2, and PDE4D1/2 are recruited to lipid rafts upon TCR and CD28 co-stimulation (117, 149). The scaffolding beta-arrestin protein has been reported to recruit PDE4 to the plasma membrane (150, 151), and the recruitment is mainly induced by CD28 stimulation (117). Moreover, the β-arrestin/PDE4 complex was shown to preexist prior to stimulation, indicating that the partners are recruited to the lipid rafts together (117). Stabilization of the β-arrestin/PDE4 complex in the lipid rafts is required for an efficient cAMP regulation by PDE4. Bjorgo and co-authors have found that recruitment of β-arrestin/PDE4 to the plasma membrane happens directly through protein kinase B (PKB) better known as Akt, with which β-arrestin and PDE4 form a complex (152, 153). Indeed, upon TCR/CD28 stimulation PI3K activity generates phosphatidylinositol-3,4,5-trisphosphate (PIP3) at the plasma membrane, which in turn directly recruits PH domain-containing PKB and, therefore, β-arrestin/PDE4 to the lipid raft (153).

Specificity of the cAMP/PKA signaling pathway is determined by generation of local gradients of cAMP controlled by PDEs and by spatially and temporally restricted activation of compartmentalized pools of PKA at different subcellular locations facilitated by AKAPs. Thus, AKAPs participate in organizing the functional complexity of cAMP signaling pathways.

## AKAPs in T Cells

A-kinase anchoring proteins bind to the regulatory subunit of PKA and ensure specificity and diversity in signal transduction by placing the enzyme close to relevant substrate (154–158). The functional importance involves the targeting of PKA to specific subcellular compartments, including the plasma membrane, nuclei, and mitochondria (159), and thereby provides spatial and temporal regulation of the PKA signaling events. Moreover, by interacting with additional signaling molecules, such as PDEs, protein kinase C (PKC), and phosphoproteins phosphatase 1 and 2B (PP1/2B) (160), AKAPs coordinate multiple signals transduction pathways and relay specific signals to downstream targets.

**Figure 2:**
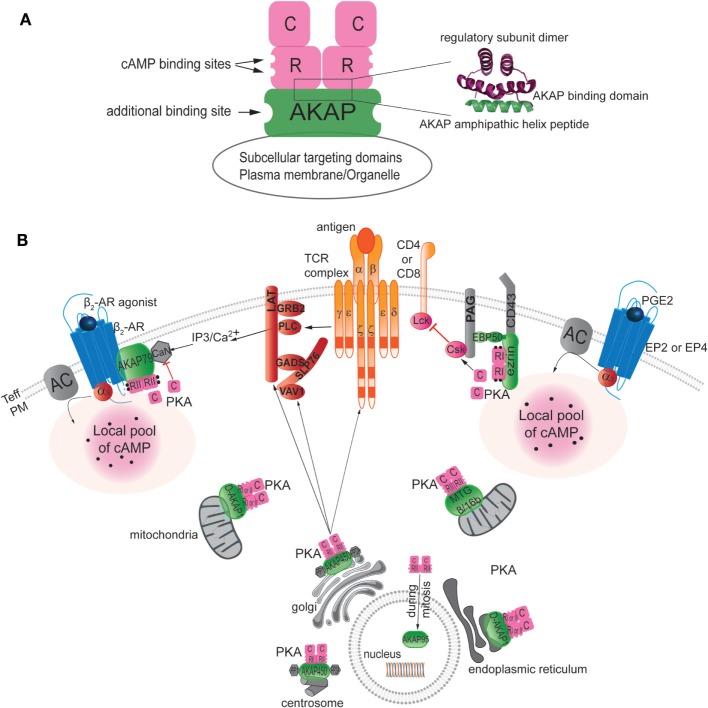
**Localization of AKAPs in T cells**. **(A)** Left: schematic diagram of an AKAP anchoring PKA through hydrophobic interaction between the amphipathic helical region of AKAP and the N-terminal dimerization region of the two R subunits of PKA. When cAMP binds to the R subunit, the C subunit of PKA is activated and released to phosphorylate nearby substrates. The AKAP signaling scaffold also typically has additional binding sites for other signaling proteins, such as kinases, phosphatases, phosphodiesterases, or potential substrates. Finally, the AKAP target the supramolecular signaling complex to the appropriate subcellular compartment via protein–protein or protein–lipid interactions. Right: ribbon representation of the NMR structure of the regulatory subunit (green) in complex with the AKAP amphipathic helix peptide (pink) [modified from (162)]. **(B)** AKAPs target PKA to specific compartments in T cells, including the plasma membrane (PM), mitochondria, endoplasmic reticulum, Golgi, nucleus, and centrosome. The same AKAP can be found in different compartments, as illustrated by the presence of D-AKAP-1 both in mitochondria and the endoplasmic reticulum and by finding AKAP450 in the Golgi and at the centrosome. AKAPs bind to specific partners and, hence, define specific supramolecular complex at discrete subcellular locales. For example, besides targeting PKA AKAP79 was shown to interact with beta 2-adrenergic receptor (β_2_AR) and calcineurin (CaN) at the plasma membrane and AKAP450 with PP1 and PP2A to the Golgi and the centrosome area. The role of each AKAP in T cells has not yet been reported. AKAP450 appears to be needed for early events as CD3, LAT, and Vav1 activation as well as late events as IL-2 production but the mechanism is still not determined. However, studies of the role of ezrin and AKAP79 have delineated their functions in downregulation of T-cell function by dampening signaling through the TCR pathway at the level of inhibition of Lck activity or by blocking IL-2 production through the inhibition of the CaN phosphatase activity, respectively.

A-kinase anchoring proteins are a structurally diverse family of functionally related proteins that comprise more than 50 members [reviewed in Ref. (161, 162)]. All the anchoring proteins contain a PKA-anchoring domain, which binds the R subunit of PKA and a unique targeting domain directing the PKA–AKAP complex to subcellular structures, membranes, or organelles (Figure 2A). AKAPs are defined as PKA type I or type II specific or dual specific depending on whether they preferentially interact with PKA type I or PKA type II or interact with both. Treating cells with the anchoring disruptor peptide (Ht31) can disrupt PKA localization. The anchoring disruptor peptide binds to the R subunits of PKA, preventing their binding to AKAPs. Several studies have illustrated that delocalization of PKA blocks its ability to respond to cAMP level increases (163, 164).

A-kinase anchoring proteins have been identified in T cells and have been shown to contribute to the maintenance of T-cell homeostasis (3, 165). AKAPs are found in lipid rafts in T cells, as well as in dendritic cells, macrophages, and likely in platelets (3, 166–170). Among the 50 members of the AKAP family, seven different AKAPs have been detected in T cells: Ezrin, AKAP79, D-AKAP1, AKAP450, MTG8, MTG16b, AKAP95, and AKAP220, but their exact and individual functional roles in T cells have not been fully elucidated (Figure 2B).

### Ezrin

Ezrin is a 78-kDa protein, which belongs to the ERM family of proteins that play structural and regulatory roles. Ezrin was originally identified as a component of sub-cortical structure underneath the cell membrane that contains an actin cytoskeleton (171, 172), and as a substrate of specific PTKs (173) [reviewed in Ref. (174)]. Ezrin and Moesin are expressed in lymphocytes (175, 176). They are cytoskeletal adaptor proteins that crosslink cell membrane and cytosolic proteins to the actin cytoskeleton and thereby govern membrane structure, its organization, and help to regulate diverse signaling routes. Ezrin has an N-terminal containing a four-point-one and ERM (FERM) domain, a central α-helical region spanning the A-kinase binding domain, AKB, linking ezrin to PKA (3, 177), and a C-terminal actin binding domain (178). The ERM family interacts with effectors of intracellular signaling either directly through the FERM domain or indirectly through adaptor molecules, such as EBP50 (179–181). In the cytoplasm, ezrin exists in a dormant form, which is unable to interact with its ligands; the binding sites for interaction partners are masked due to an intramolecular interaction between the FERM domain and the C-terminus. Phosphorylation by PKC or Rho kinase of the threonine residue, T567, at the C-terminal of ezrin, induces the conformational switch from the dormant to the active form, the intramolecular bond is released. In the active form, the N-terminal region binds to plasma membrane lipids and cytoplasmic tails of transmembrane proteins, while the C-terminal region binds to F-actin (174).

In T cells, ERM proteins control cell shape, cytokinesis, and cell adhesion (182–184) and participate in immune synapse formation (180, 185). In addition, the ERM family maintains lipid raft structures in T cells (179, 180) and contributes to control apoptosis signaling (186). Although generally described as functionally redundant, ezrin and moesin can display distinct and critical roles in the T-cell cortex during IS formation, thus promoting T-cell activation (187). Indeed, phosphorylated ezrin directly interacts and recruits ZAP-70 in the IS formation, whereas dephosphorylated moesin is removed, along with CD43, to prepare a region of the cell cortex for IS (188). The first evidence that ERM proteins play an important role in T-cell activation came precisely from studies on CD43, a large, glycosylated surface protein abundantly expressed on lymphocytes. CD43 seems acting in part as a negative regulator of T-cell activation by impeding the effective interactions of other surface receptors. Indeed, interaction with antigen-presenting cells leads to the removal of CD43 from the IS region (189, 190). ERM protein colocalizes with CD43 at the distal T-cell pole, and disruption of the interaction, either by overexpression of the FERM domain or by mutation of the relevant amino acids in CD43, leads to loss of CD43 movement and disruption of some aspects of T-cell activation.

Identified as an AKAP (177), the function of Ezrin was refined as the most important AKAP for PKA type I in T-cell lipid rafts (3). Indeed, the inhibitory effect of PKA on T-cell function is released by disruption of PKA and ezrin interaction by using specific PKA-anchoring disruptors [peptides Ht31, RI anchoring disruptor (RIAD)], which displace PKA type I from the lipid rafts (3, 191, 192). Furthermore, small interfering RNA (siRNA)-mediated knockdown of Ezrin abrogated cAMP regulation of IL-2 secretion (3), whereas reconstitution with siRNA-resistant wild-type Ezrin restored the cAMP regulation of IL-2 secretion (3, 192). Mapping studies of Ezrin reveal that the PKA RIα binding sequence is located in the α-helical region between the FERM domain and the C-terminus (3). Thus, Ezrin places PKA type I in the proximity of its TCR-proximal substrate Csk that is bound to PAG/Cbp. Moreover, ERM proteins through EBP50 have also been shown to interact with PAG/Cbp in lipid rafts (180). Together, the AKAP ezrin, EBP, and PAG/Cbp form a scaffold that holds and colocalizes PKA and Csk (3, 119, 193). Combinations of knockdown and reconstitution experiments with ezrin have demonstrated that cAMP/PKA regulation of Csk is heavily dependent on the supramolecular complex formation organized by ezrin (3, 192).

In the coming years, the role of ezrin in T cells regulation could be expanded. A new role for ezrin as an AKAP for the Connexin 43 has already been suggested by Pidoux and co-authors (124).

Connexins are a family of multiple-span membrane proteins, which construct GJ intercellular channels. Connexins have a short (~20 amino acid) cytoplasmic amino terminus and a highly variable (18 amino acids in Connexin 26, 156 amino acids in Connexin 43, and 275 amino acids in Connexin 57) cytoplasmic carboxyl terminus, which determines their overall size (194). Connexin43 (Cx43) has been described as a fundamental constituent of the immunological synapse (194), and as contributing in the regulation of proliferation of peripheral T cells (195). In Treg, Cx43 contributes not only to the formation of GJs with target cells (42), but has also been found to enhance the generation of Treg through the regulation of FoxP3+ expression (196). Besides intercellular communication, Cx43 through its C-terminal cytoplasmic domain interacts with cytoskeleton and signaling molecules, such as PKA and PKC (197). cAMP-enhanced GJ assembly has been reported to be PKA mediated (198). PKA activation increases intercellular communication, whereas PKC activation abrogates communication through GJs (199), suggesting regulatory mechanism balancing phosphorylation on GJ mediated by PKA and PKC. In human trophoblasts, Pidoux and co-authors proposed a model to explain the control and the regulation of communication through GJs. They have found that as an AKAP ezrin binds to the C-terminal part of Cx43 and delivers PKA in the vicinity of GJ (124). Indeed, upon local cAMP increase, after human chorionic gonadotropin (hCG) stimulation, PKA bound to ezrin is activated and phosphorylates Cx43. The phosphorylation of Cx43 by PKA promotes opening of the GJ and allows the passage of signal molecules. Ezrin promotes gap junctional communication by facilitating the spatiotemporal control of Cx43 phosphorylation by PKA, thereby controlling trophoblast cell fusion (124, 200).

The authors suggested that the PKA/ezrin/CX43 macromolecular complex control of GJ communication could be a general mechanism that regulates opening of Cx43 GJs in response to a cAMP increase also in others cell types (Figure 1). This study suggested an extended role of Ezrin as an AKAP in T cells, not only crucial to gather PKA type I, EBP50, PAG/Cbp, and Csk in the vicinity of TCR contributing to the control of receptor activity but also as a key regulator for the cAMP transfer from Treg to Teff, thereby contributing to Treg suppressive capacity.

### AKAP5 (AKAP79)

A-kinase anchoring protein 5 was named AKAP79 in humans and AKAP150 in rodents. AKAP79 has been found in T lymphocytes (201) and more recently in dendritic cell lipid raft (167). The C-terminus of AKAP79 has been shown to interact with the PKA regulatory subunit II, PKC, and the protein phosphatase-2B/Calcineurin (CaN) (202, 203). NFAT proteins have crucial roles in the development and function of the immune system, and are regulated by the phosphatase activity of CaN. Indeed, CaN dephosphorylates the transcription factor NFAT, which facilitates its translocation into the nucleus and the IL-2 transcription (204). The association AKAP79/CaN with the T-cell plasma membrane has been shown to inhibit CaN phosphatase activity and, therefore, the NFAT activity (201, 203, 205).

A-kinase anchoring protein 79 has also been found to bind to the beta_2_-adrenergic receptor (β_2_AR) contributing to receptor phosphorylation and signaling (206). Riether and co-authors have found that upon β_2_AR stimulation on TCR-activated CD4^+^ T cells the cellular activity of the protein phosphatase CaN was drastically reduced along with a reduction in Th1-cytokine production and T-cell proliferation. Moreover, upon β_2_AR activation, the disruption of the interaction between PKA and AKAP79 by the inhibitor peptide St-Ht31 fully blocked CaN inhibition, demonstrating that PKA–AKAP79 interaction is essential for the β_2_AR-mediated CaN inhibition. These findings suggested that upon activation β_2_AR interacts with PKA and CaN through AKAP79 forming the supramolecular complex β_2_AR/AKAP79/PKA/CaN, which leads to inhibition of the CaN activity and, therefore, blocks IL-2 production and T-cell proliferation. These findings provide evidence for a link between the β_2_AR and TCR signaling pathways and describe a novel AKAP-dependent intracellular mechanism that can lead to the downregulation of T-cell function (207).

### D-AKAP1 (AKAP149/S-AKAP84/AKAP121)

D-AKAP1 (also known as AKAP149, S-AKAP84, and AKAP121) is a member of the AKAP1 gene family (208). D-AKAP1 was reported as a dual-specific AKAP binding both RI and RII, and the N-terminus of PKA RI or RII is sufficient for its interaction with D-AKAP1 (209). D-AKAP1 is a differentially targeted AKAP, which can be localized to the mitochondrial membrane or to the endoplasmic reticulum (ER) depending on its NH2-terminal targeting motif (208). Experiments have found the presence of AKAP149 in T lymphocytes and more precisely in lipid rafts but so far no functional role was identified for AKAP149 in T-cell activation and regulation (3, 202). Lemay and co-authors have found an interaction between AKAP149 and HIV-1 reverse transcriptase in infected Jurkat T cells with a potential role in HIV-1 reverse transcription (210).

### A-Kinase Anchoring Protein 450

The scaffolding protein AKAP450 also known AKAP9, AKAP350, or CG-NAP (centrosome and Golgi localized protein kinase N-associated protein) is associated, as the name suggests, with the centrosome and the Golgi apparatus. AKAP450 has been found in T cells (211) anchoring several protein kinases (PKN and PKA RIIα) and phosphatases (PP1 and PP2A) (212, 213). Even if the mechanism is not fully understood, AKAP450 is reported to be required for T-cell activation by regulating the conformational activation of Lymphocyte function-associated antigen 1 and TCR/CD3 molecules at the immune synapse (214), which may have to do with its role in orchestrating cytoskeletal rearrangements. Indeed upon TCR activation, AKAP450 was needed for early events, such as CD3, LAT, and Vav1 activation, and late events, such as IL-2 production. AKAP450 was described as an important component of T-cell response to antigen stimulation (214).

### Myeloid Translocation Gene Family

Two members of the myeloid translocation gene family (MTG) family have been defined as AKAPs and are found in T cells: MTG8 and MTG16b. The proto-oncogene MTG8 was originally found as part of the leukemic fusion gene, AML1–MTG8 (215–217). MTG8 is expressed ubiquitously in human tissue but with varying levels of expression (high in brain, heart, and muscle and low in hematopoietic tissues) and cell-dependent localization (found in the nucleus or in the cytoplasm) (218). MTG8 was identified as a transcriptional suppressor by its tight association with the nuclear matrix (219). MTG16b, which is another MTG family member, was originally identified in patients with acute myeloid leukemia, but the normal physiological function of this protein has not been reported (220). MTG8 and MTG16b interact with the PKA RII subunit with some differences in location. Whereas MTG16b target PKA to the Golgi of T lymphocytes, MTG8 and PKARII were found in Golgi/centrosome area (202, 219). Their physiological functions as AKAPs in T cells have, however, not yet been reported.

### A-Kinase Anchoring Protein 95

A-kinase anchoring protein 95 was cloned and characterized in the rat (221) and human (222). AKAP95 specifically binds the RIIα subunit of PKA with high affinity and also has a DNA binding domain (221). The interaction between AKAP95 and PKARII is cell cycle-dependent and has only been detected during mitosis when the nuclear envelope is disassembled (222, 223) and the PKA/AKAP95 complex has a role in controlling chromosome condensation (224, 225).

The presence of AKAP95 has been reported in T cells (202) but no functional role has been identified for AKAP95 in regulating T-cell activation.

### A-Kinase Anchoring Protein 220

A 220-kDa AKAP220 was the first AKAP described able to coordinate the location of PKA and the type 1 protein phosphatase catalytic subunit (PP1c) (226, 227). AKAP220 has been found to interact with the PKA RII (2) and its presence was detected in Jurkat T cells but not in primary T cells (228).

The diversity of AKAPs in T cells and their functional role, when known, suggest critical new roles that could help to unravel the T-cell function. AKAPs, their interacting partners and appropriate targets shape the biological role of these scaffolds and supramolecular signaling complexes inside T cells; AKAPs are able to interact with PDEs, providing a route of cAMP degradation. The compartmentalization of such enzymes is crucial for the generation of intracellular cAMP gradients. The ability to form and shape intracellular cAMP pools depends on targeted PDE activity. In T cells, AKAP95, AKAP149, and MTG8/16b are in complex with PDE4A, AKAP450 with PDE4D3 (229), whereas only MTG8 is in complex with PDE7A. AKAP79 did not form a complex with either PDE4A or PDE7A (230). The specific interaction between PDE4A and selective AKAPs in T lymphocytes creates a regulatory-feedback signaling allowing localized PDE activity, compartmentalized cAMP production and PKA activity and, consequently, controlling T-cell activation with a specific pattern. This finely tuned regulatory process is not exempt from facing diverse defects, which can lead to abnormal conditions and, therefore, disease development (231, 232).

## Cyclic AMP Immunoregulation in Disease Conditions

Several human tumors and infectious diseases are characterized by high levels of intracellular cAMP (233–236). Rising cAMP concentrations have been correlated with upregulation of COX-2 levels and PGE_2_ and modulation of immune responses (16, 29, 69, 236–238). For example, T cells from HIV-infected patients contain twice as much cAMP as those of healthy controls leading to downregulation of TCR signaling and immunosuppression through an aberrant activated cAMP/PKA signaling pathway (233, 234, 239, 240).

The tumor microenvironment may foster immune tolerance by attracting and/or inducing immunosuppressive networks to escape tumor-specific immunity in favor of disease progression (241). Several studies have defined Treg as a leading player in cancer progression through a PGE_2_/cAMP-dependent suppressive ability (4, 37, 38). Patients with several forms of cancer, including gastrointestinal, lung, and ovarian tumors, have been shown to display increasing numbers of circulating and tumor-associated Treg compared with healthy controls (242–245). Moreover, Treg depletion in animal models has been shown to enhance anti-tumor responses underscoring the role of Treg to the impaired anti-tumor immunity (246–248). In addition, the percentage of Treg has been associated not only with the disease progression but also with disease outcomes. Indeed, the percentage of Treg in peripheral blood was inversely correlated with disease prognosis in patients with gastrointestinal malignancies (244). Use of COX-2 inhibitors has been shown to reverse Treg suppressive effects (38). For example, in a murine lung cancer model, inhibition of COX-2 has been found to enhance anti-tumor immune responses (4, 5). COX-2 is overexpressed in 85% of human colorectal cancers (CRCs) and approximately 50% of colorectal adenomas leading to high PGE_2_ concentrations and chronic inflammation around the cancer (249). Regular use of COX inhibitors, including aspirin reduces the incidence of CRC by 30–45% (250–253). In CRC patients, the anti-tumor immune responses of Treg are reported to be COX-2/PGE_2_/cAMP dependent and can be reversed by COX-2 inhibitor, PKA inhibitor, or Treg depletion (6). COX-2 overexpression is also correlated with the development of CRC metastases (254). Indeed in CRC patients with recurrent disease, T cell phenotyping has revealed high frequencies of COX-2 and high plasma PGE_2_ levels after surgery. Brudvik and co-authors have established strong correlation between Treg level, PGE_2_-mediated suppressive anti-tumor activity and disease recurrence (69).

Prostaglandin E_2_ plays a crucial role in the neoplastic process by stimulating tumor cell proliferation, tissue invasion, promoting angiogenesis, and by suppressing tumor cell apoptosis (255–257). Through Gs-coupled EP receptor signaling pathways, PGE_2_ expands and recruits Treg in tumor environment, which in turn suppresses T-, B-, and NK-cell immune responses, and contributes to tumor immune tolerance (38, 119, 258, 259). This combined with EP receptor activation, which triggers the cAMP/PKA/Csk signaling pathway, leads to downregulated TCR signaling and then further decreased T-cell immune function (6, 259, 260). All together these findings highlight the significant impact of the cAMP/PKA/Csk pathway on PGE_2_ control of tumor immune responses.

Along the same line, hyper-activation of the cAMP/type I PKA pathway is involved in T-cell dysfunction in immunodeficiencies. HIV-1 infection is associated with increased levels of cAMP and enhanced activation of PKA (233, 234). The HIV-1 protein gp120 functionally activates Treg by binding to CD4 and inducing enhanced AC activity and elevated intracellular cAMP levels in Treg, thereby increasing their suppressive activity on Teff (42, 261, 262). Moreover, cytokine networks have been found to be under cAMP-mediated regulation in T cells from HIV-infected patients. These findings indicated that high intracellular cAMP concentrations contribute to T-cell anergy in HIV infection. Accordingly, drugs that decrease intracellular cAMP levels may restore T-cell proliferation and cytokine networks providing a stronger antiviral response and be beneficial in the treatment of AIDS (240, 263). A similar mechanism has been found to contribute to the T-cell dysfunction in a subset of patients with common variable immunodeficiency (CVID) (264). Low level of IL-10 secreted by T cells observed in CVID patients has been related to the cAMP/PKA type I signaling. This pathway could represent a novel target for therapeutic immunomodulation in CVID (265). Similarly, in the murine AIDS (MAIDS) model induced by the murine leukemia virus, hyper-activation of the cAMP/PKA pathway, related to high level of PGE_2_, was found to contribute to severe T-cell anergy, typical feature of the pathology (238, 266). All together these findings support the idea that the cAMP/PKA pathway in T cells is a target for treating immunodeficiency diseases, chronic infections, and cancer.

Control of the cAMP/PKA pathway could help to kill tumor or infected cells, restore, or build a specific environment for robust immune responses. To this end, different strategies are possible, either upstream of the cAMP/PKA cascade by using COX-2 inhibitor, EP receptor antagonist, or downstream, blocking the intracellular cAMP/PKA cascade or its anchoring or even combining these strategies. Alone or in combination with other clinical therapeutic strategies, COX-2 could be a target to improve the efficiency of cancer and HIV treatments. *In vivo* experiments in the MAIDS model have shown that treatment with COX-2 inhibitor reduces PGE_2_ levels, reverses T-cell anergy, and thereby restores T-cell immune function (238). Moreover, combination between COX-2 inhibitors and antiretroviral treatment of HIV-infected patients has contributed toward improving T-cell proliferation and persistent immune activation. The modulation of cAMP may represent a therapeutic strategy in HIV infection in addition to antiretroviral therapy (237, 267, 268). As mentioned earlier effectiveness of selective COX-2 inhibitors has also been supported by several studies in cancer treatment and often associated with reduction of mortality rate. However, COX-2 inhibitors have also been related to serious cardiovascular events, which has resulted in interruption of long-term trials for cancer prevention (269, 270). Modulation of targets downstream of COX-2 is expected to improve the drug efficacy, specificity, and safety. Indeed, COX-2 inhibitor activity, through reduction of PGE_2_ synthesis, is not exclusive to the cAMP/PKA pathway. Actually G protein-dependent and -independent EP signaling pathways as well as crosstalk between EP signaling and parallel signaling pathways are blocked by COX inhibitor treatment. Such broad action leads to unwanted effects and call to delineate appropriate targets in order to better define exclusive inhibitors. Current knowledge has already defined several proteins required in the cAMP/PKA signaling activation, all of which could become potential targets for inhibitors, each with presumably different biological consequences. A study in a mouse model developing multiple adenomas in the intestinal tract at an early age has illustrated these potential biological differences. Indeed, whereas the anti-tumorigenic effects were correlated to COX inhibitors, the anti-proliferative effects were linked to PKA antagonism (7). These findings have identified specific chemo-protective actions related to the nature of the inhibitor and more precisely to its target and its action in the PGE_2_ signaling pathway. In a previous study, a specific PKA type I antagonist, Rp-8-Br-cAMPS, has been found to increase T-cell proliferation and restore immune responses of T cells from HIV-infected patients. These findings suggested a novel strategy in treatment of HIV infection, which would combine treatment modalities counteracting PKA type I activity and antiretroviral therapy (233, 239). Given the importance of the cAMP/PKA pathway, compartmentalized cAMP signaling and PKA activity in immune responses regulation, the targeting of AKAPs complexes for new therapeutic intervention in cancer and chronic infection has become clearly apparent. All together, these findings underscore the importance to develop agents able to specifically disrupt AKAP type I complexes.

### Disruption of AKAP Complexes in T Cell and Therapeutic Perspectives

Generation of peptides that disrupt AKAP complexes is challenging especially for therapeutic purposes. One strategy to disrupt the interaction between AKAP and PKA is to selectively displace PKA subtype from the AKAP platform with peptides that mimic the amphipathic helices domain of AKAP. Such disruptors of the AKAP complex have to be cell permeable and require high specificity and high binding affinity for their target. Most AKAPs bind avidly to the RII isoform (271), whereas others, such as Ezrin, are RI-selective AKAPs (3). A third class of AKAPs, termed dual-specific AKAPs, can bind both the RI and RII isoforms, yet their preference for binding the RII isoform strongly predominates (209). The first AKAP disruptor peptide described, Ht31, is derived from the RII-binding domain of AKAP-Lbc (AKAP13) (272). Ht31 is a peptide, which forms an amphipathic helix mimicking that found in AKAPs (273). The helix binds to the regulatory subunits of PKA disrupting localization with both RI and RII from AKAPs (274). Disruption of the AKAP–PKA interaction with Ht31 was shown to induce cytokine production (increase of IL-2, IL-4, IL-5, and IFNγ secretion) and in synergy with Ag to enhance T-cell proliferation, suggesting that PKA is necessary for maintaining T cells in a resting state. Furthermore, Ht31 treated cells were insensitive to the inhibitory effects of cAMP on IL-2 production, indicating that anchored PKA activity is necessary for cAMP-mediated inhibition of T-cell activation (165). Moreover, use of Ht31 peptide as an inhibitor of the binding between ezrin and PKA type I has shown a release of the cAMP/PKA type I-mediated inhibition on T-cell proliferation (3).

Since then, studies have identified multiple high-affinity RII-selective disruptors. In 2003, the first high potent RII inhibitor peptide, AKAP-*in silico* (AKAP-*IS*), was designed by a bioinformatics approach combined with peptide array screening (275). This peptide was shown to have higher affinity for RII as compared to Ht31 peptide. The initial peptide had limited solubility in aqueous solution and was not cell permeable. The introduction of a peptide derived from the TAT protein of the HIV-1 greatly improved cell permeability (276). A later version called SuperAKAP-*IS* was developed with high affinity for RII and almost none for RI (277). Because of structure instability, disruptor peptides may lose their cell-penetration abilities, specificity for the target or binding affinity and become more susceptible to degradation. Stabilization of their bioactive structure, thus, rapidly became a priority for cell-based assays. The all-hydrocarbon staple has emerged as one solution combining two distinct conformational stabilization strategies [reviewed in Ref. (278)]. The stabilized conformation was associated with increase in target affinity, stability against proteolysis, and robust cell-penetration while remaining safe in *in vivo* models (279–281). Using this technique, two stapled AKAP disruptor (STAD) peptides were developed: STAD-2 and STAD-3 (282). The new class of AKAP disruptors, highly cell permeable, has been shown to block interactions between AKAPs and RII and was described as a promising tool to study compartmentalized RII-regulated PKA signaling in cells.

Designing peptides for RI-selective interaction has been more challenging than for RII-selective peptides. Indeed one RI-specific disruptor peptide, PV-38, was designed from D-AKAP2 (283). In parallel, a bioinformatics analysis combined with a peptide array screening had led to the development of a peptide that binds RIα with high affinity and specificity. This high-affinity binding peptide called RIAD has been found to specifically disrupt anchoring of PKA type I from intracellular locations and inhibited type I regulation of T-cell effector function and steroid biosynthesis (191). RIAD was proposed as a tool to define anchored PKA type I signaling events and has been used extensively like in a MAIDS model described later in this review (284). Wang and co-authors also designed a series of RI-Stapled Anchoring Disruptors (RI-STADs) where two peptides, RI-STAD-1 and RI-STAD-2, were reported to have improved cell permeability and to selectively disrupt the interactions between AKAPs and PKA-RI in biochemical and cell-based assays (285).

Despite technical advances improving the specificity for PKA subtype I or II, these disruptor peptides will non-specifically inhibit all AKAP interactions with either RI or RII isoforms. Taking into account that most of cell types express at least 10–15 different anchoring proteins (2), and so far seven identified in T cells, the specificity for PKA subtype I or II is obviously not enough to specifically displace interaction between PKA and one specific AKAP. Recently, a new approach based on a phage selection procedure was employed to engineer RII sequences (R_select_) able to selectively target particular AKAP. Biochemical and cell-based experiments validated the efficacy of R_select_ mutants for AKAP2 and AKAP18 (286). Described as a new class of reagents, these genetically encoded AKAP-selective probes could help to design new compounds targeting specifically individual AKAP and to unravel the functions of different AKAP-targeted PKA. Another strategy would be to disrupt individual AKAP complexes by displacing interaction partners other than PKA, such as the substrate for PKA if that is bound to the AKAP. For example, interaction between AKAP18δ with phospholamban (PLN) was disrupted by using a short peptide derived from the PLN interaction site for AKAP18δ. This pharmacologic tool has allowed determining partners, conditions of formation, and biological consequences of the supramolecular complex formed in cardiac myocytes (287). Another possibility would be to disrupt the interaction between the AKAP targeting domain and the interaction partner providing its subcellular localization. As an example, a cell-permeant peptide of the Ezrin binding domain in EBP50 (EBP50_pep_) has been shown to displace Ezrin and reverse the cAMP/PKA-mediated inhibition of T-cell activation due to loss of PKA proximity to Csk (288). Along the same line, an inducible competing muscle-specific A-kinase anchoring protein (mAKAP) fragment (residues 585–1286) was used to displace the mAKAP from the perinuclear membrane highlighting the importance of its localization in the control of cardiomyocyte size (289). These strategies might offer higher selectivity.

Because peptides need to be administered parenterally, possess a short half-life hampered by limited stability in serum, and may generate immune responses, their potential therapeutic applications are limited. Non-peptidic agents, such as peptidomimetics and small molecules, could be one solution to counteract these drawbacks. Peptidomimetics are compounds whose essential elements mimic a natural peptide or protein in the three-dimensional space. Peptidomimetics, thus, provide a possible strategy for the modulation and regulation of AKAPs with the ability to interact with the biological target and, therefore, to displace other potential interactions without degradation. RIAD peptidomimetics were developed by adding unnatural amino acids at different positions, increasing the stability in serum while keeping their specificity to disrupt the AKAP/PKA-RI interaction (290). The RIAD peptidomimetic, RIAD-P3, was shown to limit HIV-1 viral replication and stabilize CD4 levels by disrupting AKAP/PKA-RI in human T cells and humanized mice (291). Thus, peptidomimetic research emerges as an indispensable tool of structure–activity relationships in drug discovery. Small molecules are also promising alternatives to disruptor peptides and offer several manufacturing and delivery advantages for drug discovery. Several examples have shown the ability of small molecule to disrupt protein–protein interactions (292). Small molecules interfering by orthosteric or allosteric binding have been identified (293, 294). In summary, the specificity and diversity of protein–protein interactions offer promising opportunities to develop highly selective inhibitors. However, their development requires detailed knowledge about the interaction between the two proteins (295). Displacing selected proteins from AKAP complexes could improve efficacy and specificity of anchoring disruptors with fewer side effects. This approach may lead to alternative strategies for the treatment of diseases associated with altered cAMP signaling. All together improved peptides, peptidomimetic and small molecules can help to characterize and understand molecular mechanism converging and emerging from AKAP platforms. These agents could help to define “druggable” target and alternative therapeutic strategies for the treatment of diseases associated with altered cAMP signaling.

Despite challenges to find and generate selective AKAP disruptor peptides, recent technical breakthroughs and findings from *in vitro* and *in vivo* studies strongly depict targeting protein–protein interaction as promising therapeutic strategies. Indeed, experiments using Ht31 peptides in *in vitro* experiments in T cells (3, 165) and a specific PKA type I antagonist, Rp-8-Br-cAMPS in T cells from HIV-infected patients as well as in a CRC mouse model (7, 233, 239) have underscored the major role of the AKAP, ezrin, in T-cell immune functions. Moreover, identification of an additional PKA-binding determinant, the RI specifier region (RISR), upstream of AKB in Ezrin has strengthened the role of Ezrin/PKA complex in T-cell signaling regulation. Indeed mutations in the RISR of Ezrin have been shown to perturb RI anchoring and alter the suppression of T-cell signaling through the cAMP/PKA type I/Csk pathway. The RISR was shown to act in synergy with the AKB to enhance anchoring of PKA type I (192). In an extended study, RIAD transgenic mice were generated by expressing a soluble ezrin fragment with the endogenous RISR and RIAD substituting the endogenous AKB domain under control of the lck distal promoter (284). Peripheral T cells from RIAD transgenic mice were resistant to cAMP-mediated inhibition and displayed enhanced T-cell signaling and responsiveness. Furthermore, these mice did not develop MAIDS when infected with the murine leukemia virus, as did wild-type littermates or mice infected with a mutated transgene that did not bind and displace PKA (284). These findings define the cAMP/type I PKA pathway in T cells as a putative target for therapeutic intervention through AKAP complex disruption in immunodeficiency diseases and cancer. Besides ezrin, D-AKAP1 is also involved in the progression of HIV infection. D-AKAP1 was shown to interact with the HIV-1 reverse transcriptase and to support viral replication during HIV infection (210). The full mechanism is still unknown but PKA and PDE4, key players in the cAMP signaling pathway and anchored by D-AKAP1 to mitochondria, can stimulate HIV-1 replication and infection (296–298). Hence, D-AKAP1 could affect reverse transcription through a PKA-dependent signaling pathway. Based on these results, D-AKAP1 could be another potential target in HIV therapy.

## Concluding Remarks

Cyclic AMP is a potent regulator of the immune response. Insights into molecular mechanisms causing and controlling the generation of cAMP and its propagation through the cAMP/PKA pathway are undoubtedly crucial to generate immunomodulatory agents. Several studies have assessed the efficiency of non-NSAIDs to regulate intracellular cAMP concentration and then further to control T-cell function. However, their efficiency could be impaired by lack of specificity. Indeed their early inhibitory action on PGE_2_ production blocks all EP receptor signaling pathways instead of specifically inhibiting the cAMP/PKA signaling pathway, which could create unwanted effects with biological consequences. Unraveling mechanisms surrounding PKA phosphorylation events and localization with AKAPs will hopefully support the development of more targeted therapies. Bolstered by both technological advances and learning from *in vitro* and *in vivo* experiments, AKAP disruptors emerge as essential tool, which selectively probe anchored PKA signaling and decode functions of AKAP/PKA interactions. AKAPs have crucial role in T-cell function and are involved in development and regulation of multiple chronic diseases, which make AKAPs potential targets for drug discovery. In a broader perspective, their ubiquitous nature and capacity to act as signaling hubs where multiple signals converge also offer other promising targeting perspectives.

## Author Contributions

VLW and KT wrote the article and reviewed and/or edited the manuscript.

## Conflict of Interest Statement

The authors declare that the research was conducted in the absence of any commercial or financial relationships that could be construed as a potential conflict of interest.
